# Social justice in health system; a neglected component of academic nursing education: a qualitative study

**DOI:** 10.1186/s12912-021-00534-1

**Published:** 2021-01-12

**Authors:** Hosein Habibzadeh, Madineh Jasemi, Fariba Hosseinzadegan

**Affiliations:** Faculty of Nursing and Midwifery, Urmia University of Medical Sciences, Urmia, Iran

**Keywords:** Social justice, Health equity, Education, Nursing, Qualitative study

## Abstract

**Background:**

In recent decades, increasing social and health inequalities all over the world has highlighted the importance of social justice as a core nursing value. Therefore, proper education of nursing students is necessary for preparing them to comply with social justice in health systems. This study is aimed to identify the main factors for teaching the concept of social justice in the nursing curriculum.

**Method:**

This is a qualitative study, in which the conventional content analysis approach was employed to analyze a sample of 13 participants selected using purposive sampling method. Semi-structured interviews were conducted to collect and analyze the data.

**Results:**

Analysis of the interviews indicated that insufficient education content, incompetency of educators, and inappropriate education approaches made social justice a neglected component in the academic nursing education. These factors were the main sub-categories of the study and showed the negligence of social justice in academic nursing education.

**Conclusion:**

Research findings revealed the weaknesses in teaching the concept of social justice in the nursing education. Accordingly, it is necessary to modify the content of nursing curriculum and education approaches in order to convey this core value. Since nursing educators act as role models for students, especially in practical and ethical areas, more attention should be paid to competency of nursing educators, specially training in the area of ethical ideology and social justice.

**Supplementary Information:**

The online version contains supplementary material available at 10.1186/s12912-021-00534-1.

## Background

Professional values include action standards that are accepted by group members and provide a framework for evaluating beliefs and notions affecting behavior [1]. Acquisition of professional nursing values is a prerequisite for resolving conflicts; it improves service quality and increases job satisfaction of nurses [2]. The core values accepted and presented by American Association of Colleges of Nursing (AACN) (1998) include human dignity, integrity, autonomy, altruism, and social justice [3], out of which social justice has attracted more attention in recent years. Disproportionate burden of diseases and deaths in parts of the society associated with environmental and socioeconomic factors has been recognized for decades; however, the number of documents on these issues has increased dramatically over the past 15 years [4]. The WHO Commission on Social Determinants of Health attributes these differences to social inequalities in the distribution of power, income, shelter, education, and healthcare as well as climate change, vulnerability, and other life conditions. It also prioritizes social justice as a mechanism for correcting and eliminating inequalities [5]. Social justice in the health system refers to providing equal healthcare services for all individuals, regardless of their personal characteristics [6]. The AACN defines social justice as fair treatment, regardless of one’s economic status, race, ethnicity, age, citizenship, disability, or sexual orientation [7].

Although social justice has been identified as a professional value in documents issued by reputable nursing associations such as International Council of Nurses (ICN), Canadian Nurses Association (CNA), American Nurses Association (ANA), and AACN [8], the discussion of social justice in nursing profession has always been accompanied by serious doubts and concerns [9]. In addition, nurses’ responses to social injustice have not always been admirable, and nursing profession’s poor performance originates from various factors such as unawareness [10].

Development of a professional value such as social justice is a continuous and long-term process that begins with professional nursing education and continues throughout years of nursing practice. Education plays a key role in acquiring professional values [11]. Students, educators, faculties, clinical and educational experiences, and individual values are among the most important components of learning and development of professional values [12]. It is very important to train highly skilled and qualified nurses to provide necessary care for heterogeneous populations in today’s ever-changing demographic prospect. Nursing students must understand their responsibility for poplulation health issues and social factors affecting health (eg, world hunger, environmental pollution, lack of access to health care, violation of human rights, and inequitable distribution of health care resources, including nursing services) and in this regard acquire the necessary knowledge and skills [13].

To institutionalize the concept of social justice in nursing students, especially in developed countries, measures have been taken in the area of education, which include modifications made to nursing curriculum and education approaches [14]. For instance, simulation is a one of new methods utilized for teaching this concept [15]. Since the mid-2000s, there has been an increase in tendency towards online learning [16], co-curricular experiences [17], and digital storytelling [18] in order to promote students’ understanding of social justice issues. Nevertheless, some studies have addressed the weaknesses of nursing curriculum in teaching social justice [19, 20] and have attributed nurses’ inability in pursuing social justice to their poor scientific and practical competencies [21]. Although several quantitative and qualitative studies have been conducted in recent decades to institutionalize the concept of social justice among nursing graduates [22–25], academic nursing education has unfortunately failed to train competent nurses who seek information and training on social justice. Considering the importance of this subject, a qualitative approach [26] was adopted to provide an in-depth understanding of social justice based on the realistic results derived from the participants’ real experiences. Therefore, in this study, the experiences of nursing educators and students in identifying the main factors for teaching the concept of social justice in nursing education program were analyzed.

## Method

### Study design and setting

This qualitative study was conducted using a conventional content analysis method. The participants were recruited from three nursing faculties (Urmia, Tabriz, and Tehran) and two teaching hospitals of Tehran (Motahari Hospital) and Urmia (Talegani Hospital) in Iran. These cities were selected due to their large size and forerun in educational, clinical, and social nursing activities.

### Study participants

In view of the objective of the study - identify the main factors for teaching the concept of social justice in the nursing curriculum - we initially selected nursing educators by purposive sampling method. Nursing educators who had more than 5 years of service experience and among the prominent educators with activity in nursing institutions that involved in developing social justice were selected. The data from the study then led us to students and clinical nurses. Among the students, the final year undergraduate students, exemplary and active in social fields, and among the nurses, those with more than 2 years of service experience, accepted by the system professionally and actively in the field of social justice, such as voluntary activities in public health promotion, were selected for the interview.

The participants included 6 men and 9 women with the mean age of 39.07 ± 12.92 years old and mean work experience of 20.00 ± 7.22 years. Out of all the participants, 5 individuals had PhD, whereas 2 had Master’s degrees; the rest had Bachelor’s degrees in nursing. In total, 7 individuals were nursing educators, 2 individuals were clinical nurses, and 4 individuals were nursing students (Table 1).

**Table 1:** Demographic Characteristics of the Participants

No.	Education	Work experience	Position	City
1	PhD	28	Faculty member	Urmia
2	PhD	25	Faculty member	Urmia
3	Master’s	28	Faculty member	Urmia
4	PhD	24	Faculty member	Tabriz
5	PhD	22	Faculty member	Tabriz
6	Bachelor’s	N/A	Student	Urmia
7	Bachelor’s	N/A	Student	Urmia
8	Master’s	10	Faculty member	Urmia
9	PhD	11	Faculty member/Policymaker	Tehran
10	Bachelor’s	20	Clinical nurse	Urmia
11	Bachelor’s	12	Clinical nurse	Tehran
12	Bachelor’s	N/A	Student	Urmia
13	Bachelor’s	N/A	Student	Urmia

### Data collection

The data were collected using in-depth, semi-structured individual interviews conducted at the times and in the places selected by the participants (mainly at nursing faculties). Each interview lasted for 30–90 min; they were audio recorded upon the participants’ permission and transcribed verbatim. All the 13 interviews were conducted by the research team (FH, MJ, and HH) between February and November 2019. The participants were asked questions about their experiences of (learning/teaching) social justice issues. Considering the abstract nature of the research subject, the researchers raised more objective questions. For instance, the educators were asked to “describe their experiences of modification to the curriculum to cover social justice issues”, whereas the students were asked to “describe their experiences of social justice-based practices during internships”. In addition, to better identify factors affecting social justice education in nursing, the educators and students were asked questions such as “Considering your experiences, what factors have affected your engagement in social justice in education?” and “How do you describe education approaches adopted by educators for teaching social justice?”, respectively. (See Additional file 1 for details). The researchers continued the interviews until the data were completely saturated, i.e. when no new idea, concept, or category was derived from the final interviews.

To better relate to the environments of the study and the participants and analyze the data realistically, the researchers also used field notes. Field notes are a brief summary of the observations made while collecting data. This is not limited to a particular type of activity or behavior and assesses the non-verbal behaviors of the participants and their interactions with others. It also depicts a picture of a social position. In this study, field notes also made a detailed presentation of the situation in the right place immediately after the interview and provided the opportunity to confirm the psychological and emotional reactions of the participants. For example, attending the emergency ward of one of the teaching hospitals in Urmia city and observing nursing education in the clinical environment led to a field note focusing the training on the clinical procedures that confirm the insufficient educational content and lack of attention to social justice in nursing education.

### Data analysis

After the data were collected, they were analyzed using the conventional content analysis approach. For this purpose, Grundheim and Lundman’s (2004) method was adopted [27]. In this method, an entire interview is regarded as an analysis unit involving notes that must be analyzed and coded. The researchers listened to the interviews for several times and transcribed the recorded interviews verbatim. The paragraphs, sentences, and words were considered meaning units. A meaning unit is a set of words and sentences that are related to each other in content and are categorized based on their content and context. The texts were reviewed several times to highlight words containing key concepts or meaning units and extract the initial codes. The codes were then reviewed several times in a continuous process from code extraction to labeling. Similar codes were merged, categorized, and labeled and the subcategories were determined. The extracted subcategories were finally compared and merged (if possible) to form the main categories.

### Assessing data accuracy and stability

Guba and Lincoln’s (1986) criteria were used to ensure the accuracy and stability of the research data. The credibility of the data was assessed using member-checking and prolonged engagement techniques. For member-checking technique, the participants reviewed the content of the interview and the resulting codes to ensure the accurate meaning and for really reflecting their experiences. The data were also assessed by an external researcher (peer debriefing). To ensure the dependability, data collection methods, interview, taking notes, coding, and data analysis were expressed in detail in order to make judging by the external auditor (external auditing). In order to achieve confirmability, the audit trail method was used, so that all stages of the research, especially the stages of data analysis and the results, were provided to checking of two expert colleagues in the field of qualitative research. The transferability of the findings was also established by providing a rich description of the research report and the content of the interviews was represented by the selected quotations from the participants [28].

### Ethical considerations

The participants were selected after the approval of Ethics Committee of Urmia University of Medical Sciences and the necessary permissions (Code: IR.UMSU.REC.1397.223) were granted. Prior to the interviews, the participants were informed about their anonymity, confidentiality of their information, the research method and objectives, and their right to leave the study at will. The participants also signed informed consent forms.

## Results

Classification of the interviews showed that three sub-categories of “insufficient educational content”, “limited competency of nursing educators”, and “inappropriate education approaches” led to the emergence of the main category called “social justice; a neglected component of academic education” (Table 2).

**Table 2:** Categories, Subcategories, and Codes Extracted from the Interview Analysis

Core Category	Subcategories (1)	Primary concepts	Open Codes
**Social justice; a neglected component of academic nursing education**	**Insufficient educational content**	**Lack of attention to the issue of social justice in nursing curriculum**	Deficiencies in academic courses provided on nursing ethics Deficiencies in academic courses provided on professional rights Insufficient attention to social determinants of health in education Lack of educational courses on culture-oriented care Insufficient attention to the topic of community-based care
		**Discontinuity in presenting courses on ethical values**	Presenting ethics course only for Bachelor’s program Failing to present ethics course for nursing students at all programs Failing to adequately repeat the discussed ethical topics presented to nursing students
		**Allocating most nursing courses to medical issues and clinical care**	Putting unnecessary emphasis on biological health factors Allocating a large number of courses to medical issues Focusing educations on clinical care
	**Limited competency of educators**	**Insufficient capabilities of educators in teaching social justice issues**	Insufficient knowledge of educators about ethical values Insufficient experience in teaching social justice issues Insufficient experience in social justice due to lack of regular attendance in clinical and social settings
		**Inappropriate value perspectives of educators in developing social justice**	Discussing social justice issues based on personal beliefs Placing little importance on professional ethics Believing that nurses cannot play an effective role in justice promotion
	**Inappropriate education approaches**	**Focusing on traditional education approaches**	Use of teacher-based approaches in teaching ethical issues Concentration on lecturing approach Rare use of group discussions in teaching ethical issues
		**Weakness in using affective learning approaches**	Ignoring students’ attitudes towards the issue of social justice Paying less attention to changes occurring in students’ behaviors following an educational course Failing to prepare a proper scenario for teaching ethical issues Paying less attention to self-awareness and self-reflection techniques Failing to encourage students to improve their critical thinking skills

### Social justice; a neglected component of academic education

Proper education plays a major role in training justice-seeking nurses. Social justice and its importance in healthcare are constituents of the nursing syllabus. Paying more attention to this issue in practical and objective areas of education by educators can influence students’ thoughts, attitudes, and behaviors to pursue justice in health systems. However, Iran’s education system has unfortunately failed to promote justice because of insufficient educational content, limited competency of nursing educators, and inappropriate education approaches.

### Insufficient educational content

Development of a comprehensive nursing curriculum, especially on ethical issues such as social justice, could substantially contribute to the preparation of socially and morally conscious nurses who are able to make significant changes in the public health at local, national, and international levels. In this study, the participants highlighted some weaknesses in the content of the existing nursing curriculum such as lack of attention to social justice, discontinuity in presenting courses on ethical values, and allocating most of the nursing courses to medical issues and clinical care.

#### Lack of attention to social justice in nursing curriculum

Social justice is a core nursing value which plays a significant role in promoting justice by nursing students and nurses. However, according to the participants, it has unfortunately been neglected in the existing nursing curriculum. In this regard, one participant stated,

“In the fourth semester, we studied a course on nursing ethics. I think there was no discussion on social justice because I don’t remember anything about this topic” (Participant No. 7/Nursing Student).

Regarding the importance of teaching social determinants of health, another participant stated,

“I was not aware of the importance of social issues in health until I participated in a workshop called ‘Social Justice in Health’. It really changed my beliefs and broadened my perspective” (Participant No. 10/Clinical Nurse).

#### Discontinuity in presenting courses on ethical values

Values are major components of the nursing profession. The institutionalization and development of professional values such as social justice contribute significantly to the future of this profession. The few number of courses presented on ethical values and discontinuity in the presented courses (for instance, no course on ethical values is provided for post-graduate students) were major items mentioned by the participants. In this regard, one of the participants stated,

“When students are repeatedly reminded of the importance of a value, they will realize its importance and the value will be institutionalized in them. We partially studied professional values and social justice issues in the fourth semester of our undergraduate courses; however, no similar course was provided for us afterwards during the Master’s program” (Participant No. 5/ Faculty Member).

Or another participant stated:

“We cannot deny that the ethical issues have been institutionalized in our professional graduates to some extent. But, these issues are not worked on in a principled and scientific manner and that there is no constant focus on them. After all, the effect of the hidden curriculum has been more prominent.”(Participant No.1/Faculty Member).

#### Allocating most of nursing courses to medical issues and clinical care

Diseases and clinical care are among the most fundamental parts of theoretical and practical training provided for nursing students; however, due to the multi-dimensional nature of the nursing profession, special attention should be paid to other dimensions as well. According to the research results, the existing nursing curriculum focuses mainly on transferring knowledge and skills associated with physical and routine care. One participant expressed,

“Most of our courses were related to various diseases and nursing care, and educators rarely talked about ethical and legal issues during their lectures” (Participant No. 6/ Nursing Student).

Another participant stated the reasons for the focus of nursing education on the physical and caring dimensions:

“Well, when we see that our graduates have problems in providing quality clinical care, we also have to do more in the field of clinical care.”(Participant No.3/ Faculty Member).

### Limited competency of nursing educators

Educators play an undeniable role in training competent nurses through institutionalizing beliefs and behaviors. Using proper teaching and behavioral approaches, educators can improve students’ critical thinking skills and prepare them to promote justice in health systems. According to the participants, insufficient competency of nursing educators in teaching social justice issues and inappropriate value perspectives of educators in developing social justice were the main properties of this category.

#### Insufficient capabilities of educators in teaching social justice issues

Educators must be equipped with sufficient scientific, practical, and ethical capacities in order to effectively institutionalize the concept of social justice in students. According to the participants, nursing educators’ insufficient knowledge and experience about social justice issues make it difficult for them to transfer such knowledge to their students. One participant said,

“When I was a student, I once informed my educator about the unjust patient admission procedure in the surgical department. Yet, my educator recommended me to do what the head nurses would say. I did not see the necessary authority in my educator to establish justice”

(Participant No. 11/ Clinical Nurse).

Low presence of nursing educators in clinical and community settings is also one of the factors that, according to the participants, has contributed to this problem.

“Unfortunately, our professors are so involved in education and research, especially to promote themselves, that they do not have the opportunity to address social issues.” (Participant No.9/ Faculty Member).

#### Inappropriate value perspectives of educators in developing social justice

The participants highlighted the important role of nursing educators’ ethical perspectives in promoting the quality of education and training qualified nurses who would provide services tailored to the needs of the society. They also argued that ethical values could help educators establish and expand social justice in health systems. According to the results, most of the educators had undesirable value perspectives on establishing social justice in the area of health. In this respect, participant no. 5 stated,

“When a nurse has no right to make any decisions in a healthcare system, what can I say to the student about social justice?” (Participant No. 5/ Faculty Member).

Or another participant stated:

“My main responsibility is to transfer knowledge in the field of nursing and I think ethics should be taught by educators in medical ethics.” (Participant No.2/ Faculty Member).

### Inappropriate education approaches

Education approaches are considered an essential part of the educational structure and play a key role in transferring ethical values such as social justice to students. Given the abstract nature of social justice, choosing the best education approach could help educators resolve complicated problems during teaching in order to institutionalize professional values and beliefs. According to the findings, educators adopt poor education approaches to transfer ethical values such as social justice and self-awareness to students. In this regard, focusing on traditional education approaches and using insufficient affective learning approaches were cited by the participants.

#### Focusing on traditional education approaches

Undoubtedly, lecturing is one of the most widely used education approaches; however, this traditional method is very ineffective in teaching abstract concepts such as social justice. According to the participants, educators mostly use lecturing approach to teach social justice issues and students are rarely involved in the teaching process. One participant argued that educators mainly use teacher-centered approaches in ethical discussions, stating,

“We (the students) had no active role in the professional ethics class. The educator spoke on relevant topics based on the availed syllabus and provided some examples of clinical ethical issues. However, I think that educators must discuss social justice issues with students to help them visualize and understand cases of injustice and discuss appropriate reactions in such situations” (Participant No. 13/ Nursing Student).

Another participant stated this:

“The predominant teaching method in professional ethics classes has been lecturing. Every now and then, there was some discussions in between, but it was very rare. Other nursing educators were also using the lecture method when talking about ethics” (Participant No. 7/ Nursing Student).

#### Using insufficient affective learning approaches

The use of affective learning strategies such as reflective activities and simulations leading to emotional responses plays an important role in creating self-reflection and transferring professional knowledge and skills to nursing students. However, based on the participants’ experience, affective learning approaches are not used effectively and systematically in teaching ethical issues such as social justice. In this regard, one participant stated,

“Since there are too many topics on professional ethics, we (educators) can only convey basic issues to students and it is difficult for us to adopt other learning strategies such as the affective approach” (Participant No. 4/ Faculty Member).

The same participant further stated:

“Now, in the professional ethics class, I do my best to teach the content with a combination of methods. For example, we have formed a group for medical students in the cyberspace (WhatsApp) and asked students to express the issues and questions of clinical ethics. They should raise it there because there is no time in the classroom for these issues. However, we have not performed the same for nursing students yet” (Participant No. 4/ Faculty Member).

## Discussion

According to the research findings, social justice in a health system is a neglected component of academic nursing education due to factors including insufficient educational content, limited competency of nursing educators, and inappropriate education approaches. These factors were introduced as the main research subcategories in this study.

Some weaknesses were observed in the content of nursing curriculum, which is an main factor in promoting professional nursing values such as social justice in nursing students. Lack of attention to the issue of social justice in nursing curriculum has also been mentioned in other studies [13, 20]. Based on the participants’ experience, most of the nursing courses are allocated to medical issues and clinical care. According to Thurman, clinical specialties have received the main focus of nursing curriculum, whereas little attention has been paid to social justice issues [21] . This problem can be attributed to the poor performance of nurses in clinical care. The participants also believed that there was discontinuity in presenting courses on ethical values because the professional ethics course was presented only to undergraduate students. This issue disrupts the proper institutionalization of ethical values such as social justice in nursing students. Frenk et al. believe that the preparation of healthcare professionals to address current healthcare inequalities and challenges has been slowed down by obsolete, fragmented, and static curriculum [29]. In addition, Rozendo et al. highlighted inconsistencies in terms of presenting social justice-related issues in nursing curricula and argued that there was little material on social justice in post-graduate nursing programs [14].

Nursing educators’ competencies also affect teaching social justice issues. In today’s rapidly-changing world facing numerous crises, experienced educators play a significant role in training qualified nurses equipped with various skills enabling them to create social development. Accordingly, Read et al. highlighted the critical role of nursing educators in institutionalizing fundamental principles of social justice and health equity in students [30]. According to Ellis, educators should shift nursing students’ learning and thinking attitudes from individualism to community-centered frameworks and from tertiary (reactionary) to primary (preventive) care approaches [31]. However, unfortunately, the research findings indicated that nursing educators are not sufficiently qualified to teach and institutionalize social justice in students. In this regard, educators’ insufficient knowledge and experience in teaching social justice issues were highlighted by the participants. Borhani et al. found that ethical knowledge of nursing educators determined their students’ professional ethics competencies [32]. Akbas et al also argued that nursing educators’ knowledge and skills were the first and most important factors affecting their success in teaching issues of professional ethics [33]. As mentioned by the participants, inappropriate value perspectives of educators in developing social justice was another weakness of nursing educators. The significant impact of educators’ perspectives on teaching ethical values such as social justice has also been emphasized by Parandeh et al. [12].

Education approaches adopted to present and convey ethical values to students are of high importance. In this regard, Einhellig discussed the ineffectiveness of traditional approaches such as lecturing in institutionalizing social justice in nursing graduates and outlined the benefits of affective learning approaches [19]. According to the findings, lecturing is the dominant approach used to teach social justice in Iran’s nursing faculties, which is an inefficient teaching approach, as suggested by the research literature. This is probably due to the large number of students and limited time allocated to each academic course. While cognitive learning approaches rely on principles and concepts, affective learning approaches support the integration of knowledge with emotions, attitudes, and personal beliefs [34]. Neumann found that affective education approaches could enhance students’ understanding and use of ethical values [35]. Einhellig highlighted that nursing faculties need to use various strategies with a focus on behavior changes in order to successfully institutionalize the concept of social justice in nursing graduates [24].

### Limitations

The findings of the present study were limited to factors affecting education of social justice in the nursing curriculum in the health system in the culture of Iran. Other limitations of this study was the consideration of the three nursing faculties and two teaching hospitals in Iran. As such, it may not be a representative of the experiences of all the nursing profession members in Iran. Limitations of our study proposed the need for conducting further studies with larger and mixed groups and in different cultures.

## Conclusion

The research findings provided researchers with an insight into the weaknesses of nursing curricula, educators, and education approaches in social justice development in Iran. It seems that more attention must be paid to professional values and social determinant of health in nursing curricula in order to train justice-seeking nurses with a sense of responsibility. Educators play a prominent role in training competent individuals who are aware of and sensitive to social issues and inequalities. It is necessary to change the education approaches adopted by nursing educators in order to institutionalize the concept of social justice in students. After changing the content of nursing curriculum and applying different education approaches, future studies can focus on the impact of such changes on social development and social justice promotion.

## Supplementary Information

**Additional file 1.**

## Data Availability

The interview dataset generated and analysed during the current study are not publicly available due to promises of participant anonymity and confidentiality. However, on reasonable request the data could be available from the corresponding author. All applications should be sent to Hosseinzadegan.f@umsu.ac.ir. All requests will be answered within a maximum of 1 month by email.

## Abbreviations

WHO: World Health Organization
AACN: American Association of Colleges of Nursing
ICN: International Council of Nurses
ANA: American Nurses Association
CNA: Canadian Nurses Association

## References

[1] Kaya H (2017). Personal and professional values held by baccalaureate nursing students. Nurs Ethics.

[2] Tehranineshat, B., C. Torabizadeh, and M. Bijani, A study of the relationship between professional values and ethical climate and nurses' professional quality of life. Int J Nurs Sciences. 2020;7(3):313-19. 10.1016/j.ijnss.2020.06.001 PMC7424154 32817854

[3] American Association of Colleges of Nursing, The essentials of baccalaureate education for professional nursing practice. Washington, DC: American Association of Colleges of Nursing; 1998.

[4] Perry DJ (2017). Exercising nursing essential and effective freedom in behalf of social justice: a humanizing model. ANS Adv Nurs Sci.

[5] Plamondon KM, et al. The integration of evidence from the commission on social determinants of health in the field of health equity: a scoping review. Crit Pub Health. 2020;30(4):415–28.

[6] Institute of Medicine, The future of nursing: Leading change, advancing health. Committee on the Robert Wood Johnson Foundation Initiative on the Future of Nursing. Washington, D.C.: National Academies Press; 2011. 24983041

[7] Davis RK, et al. Social justice as an expression of caring through holistic admissions in a nursing program: a proposed conceptual model. Nurs Forum. 2020;55(4):723–9. 10.1111/nuf.12489 32720314

[8] Matwick AL, Woodgate RL (2017). Social justice: a concept analysis. Public Health Nurs.

[9] Browne AJ, Reimer-Kirkham S (2014). Problematizing social justice discourses in nursing. Philosophies and practices of emancipatory nursing: Social justice as praxis.

[10] Walter RR (2017). Emancipatory nursing praxis: a theory of social justice in nursing. Adv Nurs Sci.

[11] Parvan K, Hosseini F, Zamanzadeh V. Professional Values from Nursing Students' Perspective in Tabriz University of Medical Sciences: a pilot study. Iran J Nurs (2008-5923). 2012;25(76):69–82.

[12] Parandeh A (2015). Factors influencing development of professional values among nursing students and instructors: a systematic review. Global J Health Sci.

[13] Waite R, Brooks S (2014). Cultivating social justice learning & leadership skills: a timely endeavor for undergraduate student nurses. Nurse Educ Today.

[14] Rozendo CA, Santos Salas A, Cameron B (2017). A critical review of social and health inequalities in the nursing curriculum. Nurse Educ Today.

[15] Menzel N, Willson LH, Doolen J (2014). Effectiveness of a poverty simulation in second life®: changing nursing student attitudes toward poor people. Int J Nurs Educ Scholarsh.

[16] Breen H, Jones M (2015). Experiential learning: using virtual simulation in an online RN-to-BSN program. J Contin Educ Nurs.

[17] Davis JN, Sullivan K, Guzman A (2018). Catalyst for growth: the implications of co-curricular experiences for nursing education. J Nurs Educ.

[18] LeBlanc RG (2017). Digital story telling in social justice nursing education. Public Health Nurs.

[19] Einhellig K, Hummel F, Gryskiewicz C (2015). The power of affective learning strategies on social justice development in nursing education. J Nurs Educ Pract.

[20] Canales MK, Drevdahl DJ. Social justice: from educational mandate to transformative core value, in Philosophies and practices of emancipatory nursing: Social Justice as Praxis. New York, NY: Routledge; 2014. p. 155–74.

[21] Thurman W, Pfitzinger-Lippe M (2017). Returning to the profession's roots: social justice in nursing education for the 21st century response. Adv Nurs Sci.

[22] Torres-Harding SR, Meyers SA (2013). Teaching for social justice and social action. J Prev Interv Community.

[23] Groh CJ, Stallwood LG, Daniels JJ (2011). Service-learning in nursing education: its impact on leadership and social justice. Nurs Educ Perspect.

[24] Einhellig K, Gryskiewicz C, Hummel F (2016). Social justice in nursing education: leap into action. J Nurs Care.

[25] Hellman AN (2018). Understanding poverty: teaching social justice in undergraduate nursing education. J Forensic Nurs.

[26] Speziale HS, Streubert HJ, Carpenter DR. Qualitative research in nursing: advancing the humanistic imperative. 5 th edition. 2011: Wolters Kluer: Lippincott Williams & Wilkins; 2011.

[27] Graneheim UH, Lundman B (2004). Qualitative content analysis in nursing research: concepts, procedures and measures to achieve trustworthiness. Nurse Educ Today.

[28] Lincoln YS, Guba EG (1986). But is it rigorous? Trustworthiness and authenticity in naturalistic evaluation. New Dir Prog Eval.

[29] Frenk J (2010). Health professionals for a new century: transforming education to strengthen health systems in an interdependent world. Lancet.

[30] Read CY, Betancourt DMP, Morrison C (2016). Social change: a framework for inclusive leadership development in nursing education. J Nurs Educ.

[31] Ellis S (2013). The existing intersection of social justice and nursing.

[32] Borhani F, et al. Professional Ethical Competence in nursing: the role of nursing instructors. J Med Ethics Hist Med. 2010;3(3):1-8. PMC3714123 23908738

[33] Akbas M, Kadioglu S, Tuncer I (2017). Ethics in nursing education from the viewpoints of Turkish nursing educators. Int J Educ Sci.

[34] Holt KM (2017). Affective domain learning in high-fidelity simulation: students’ perspectives.

[35] Neumann JA, Forsyth D (2008). Teaching in the affective domain for institutional values. J Contin Educ Nurs.

