# Supplementary material for: Social justice in health system; a neglected component of academic nursing education: a qualitative study
Source: BMC Nurs. 2021 Jan 12;20:16. doi: 10.1186/s12912-021-00534-1 (PMC7802265; doi:10.1186/s12912-021-00534-1)
Supplement: Supplementary file 1 — Additional file 1. [file 12912_2021_534_MOESM1_ESM.docx]

**Additional File 1:**

**Interview guide**General information
Age:
Sex:
Level of education:
Academic rank:
Occupation:
Service location:
Years of service:

**Interview Guide Questions for Nursing Educators**

• Please discuss transferring the ethical values of the nursing profession to students.
• Tell me about the methods used to institutionalize the ethical values of the profession.
• Explain the training strategies employed to teach the ethical values of the profession.
• Tell me how students are evaluated on issues related to nursing ethics.
• Please explain how you convey the concept of social justice to students.
• Share your experiences in teaching social justice and changing student behaviors.

**Interview Guide Questions for Nurses**
• What steps do you take to establish social justice?
• Based on your experience, what factors influence your participation in justice activities?
• Share your educational experiences in the field of ethical values of the profession, including social justice, from the beginning of your career.

**Interview Guide Questions for Nursing Students**• Share your teaching-learning experiences related to professional ethics.
• What are the effective methods of teaching professional ethics? Please elaborate.
• Please explain how the training provided for social justice was.
